# Impact of neutrophil-to-lymphocyte ratio on periprocedural myocardial infarction in patients undergoing non-urgent percutaneous coronary revascularisation

**DOI:** 10.1007/s12471-016-0850-6

**Published:** 2016-06-08

**Authors:** M. Verdoia, A. Schaffer, L. Barbieri, G. Di Giovine, G. Bellomo, P. Marino, H. Suryapranata, G. De Luca

**Affiliations:** Division of Cardiology, Azienda Ospedaliera-Universitaria Maggiore della Carità, Eastern Piedmont University, Novara, Italy; Division of Clinical Chemistry, Azienda Ospedaliera-Universitaria Maggiore della Carità, Eastern Piedmont University, Novara, Italy; Division of Cardiology, UMC St Radboud, Nijmegen, The Netherlands

**Keywords:** Neutrophil/lymphocyte ratio, White blood cells, PCI, Periprocedural myocardial infarction

## Abstract

**Background:**

Pro-thrombotic conditions importantly influence myocardial perfusion and procedural results after percutaneous coronary intervention (PCI). The neutrophil-to-lymphocyte ratio (NLR) has emerged as a predictor of cardiovascular events and of long-term prognosis, especially in ST-elevation myocardial infarction patients undergoing primary PCI. The aim of our study was to evaluate the role of NLR on periprocedural myocardial infarction (MI) in patients undergoing non-urgent PCI.

**Methods:**

In a consecutive cohort of 1542 patients undergoing PCI, myonecrosis biomarkers were determined at 6, 12, 24 and 48 hours post-procedure. Patients were divided into quintiles according to NLR values. Periprocedural myonecrosis was defined as a troponin I increase of 3 times the upper limit of normal or as 50 % of an elevated baseline value, whereas periprocedural MI was defined as a CK-MB increase of 3 times the upper limit of normal or 50 % of baseline.

**Results:**

Higher NLR was related to age, established risk factors and cardiovascular history. NLR was associated with severe coronary artery disease (*p* = 0.009), tighter stenosis (*p* < 0.001), coronary calcifications (*p* = 0.005), intracoronary thrombus or thrombectomy use (*p* < 0.001), TIMI flow pre- and post-PCI (*p* < 0.001), and inversely to restenosis (*p* = 0.04) and use of a drug-eluting stent (*p* = 0.001). NLR did not influence the occurrence of myonecrosis (*p* = 0.75; adjusted OR (95 % CI) = 0.99 (0.63–1.54), *p* = 0.96), but was associated with a higher occurrence of periprocedural MI, even after correction for baseline differences (*p* = 0.03; adjusted OR (95 % CI) = 1.33 (1.02–2.3), *p* = 0.02), with NLR ≥ 3 best predicting the risk of periprocedural MI at the receiver operating characteristic curve analysis.

**Conclusion:**

In patients undergoing non-urgent PCI, a higher NLR increases the risk of periprocedural MI, especially for values ≥ 3.

## Background

Technological improvements and the introduction of new, more potent, antithrombotic therapies [1–3] have allowed an escalation in the complexity of percutaneous coronary interventions (PCI), therefore enhancing the risk of periprocedural complications [4, 5].

In fact, many patients still suffer a periprocedural myocardial infarction (MI), especially as a consequence of flow-limiting complications, although a myocardial injury can also occur silently, due to the formation of leukocyte and platelet plugs in the microvascular circulation, preventing myocardial perfusion and potentially negatively influencing the prognosis [4–6]. Thus, white blood cells and inflammatory status can be involved in these events, enhancing pro-thrombotic response and platelet reactivity.

The neutrophil-to-lymphocyte ratio (NLR) has recently emerged among the markers of inflammation, being an inexpensive, easy to obtain parameter, which can improve the risk stratification of patients with cardiovascular disease [7]. In fact, it has been associated with arterial stiffness and with the indicators of the extent of coronary artery disease [8, 9], and moreover with thrombus formation in acute coronary syndromes [10]. In these settings NLR has been suggested to influence short-term and long-term outcome, especially in patients with ST-segment elevation myocardial infarction (STEMI) undergoing primary PCI [11, 12]. However, no study has so far evaluated its impact in patients undergoing non-urgent percutaneous coronary revascularization. The aim of our study was, then, to assess whether NLR can influence the risk of periprocedural MI in patients undergoing non-urgent PCI.

## Methods

We included patients undergoing coronary angioplasty at Ospedale “Maggiore della Carità” from May 2007 to January 2013 for both elective indications or acute coronary syndromes (unstable angina/NSTEMI). NSTEMI patients were defined by the presence of chest pain and cardiac biomarkers elevation above the upper limit of normality (0.04 µg/l for troponin I and 5.00 µg/l for CK-MB, respectively) and underwent elective coronary angiography after pharmacological stabilisation, but not STEMI and unstable patients requiring urgent angioplasty.

Diabetes mellitus was defined as one of the following: 1) previous diagnosis, 2) specific treatment of diabetes (oral or insulin), 3) fasting glycaemia > 126 mg/dl in at least two repeated determinations, 4) or HbA1c > 6.5 %. Hypertension was defined as a systolic pressure > 140 mmHg and/or diastolic pressure > 90 mmHg or if the individual was taking antihypertensive medications [13].

The study was approved by our local ethics committee. In accordance with the guidelines, all patients received a bolus of an antiplatelet adenosine diphosphate antagonist at the time of hospitalisation or before angioplasty. Patients were clinically followed up to hospital discharge.

## Biochemical measurements

Blood samples were drawn at admission (following a fasting period of 12 hours) in patients undergoing coronary angiography for elective or acute indications. Glucose, creatinine, HbA1c and lipid profile were determined by standard methods. The total and differential white blood cell count was measured in a blood sample collected in tri-potassium EDTA (7.2 mg) tubes. These blood samples were analysed within 2 hours of venepuncture by an automatic blood counter (Sysmex XE-2100) used for whole blood analysis. The neutrophil/lymphocyte ratio was measured for each patient. Cardiac biomarkers (troponin I and CK-MB) were measured at baseline, before coronary revascularization, and later at 6, 12, 24 and 48 hours after PCI.

## Coronary angiography and PCI

Coronary angiography was routinely performed by the Judkins technique using 6‑French catheters. Quantitative coronary angiography was performed by experienced interventional cardiologists using an automatic edge-detection system (Siemens Acom Quantcor QCA, Erlangen, Germany) [14]. Coronary angioplasty was performed with standard techniques. All patients received a bolus of unfractionated heparin during coronary angiography (80 U/kg) and if needed additional heparin was administered during the procedure according to the activated clotting time. An oral loading dose of an adenosine diphosphate antagonist (600 mg clopidogrel or 180 mg ticagrelor) was given before PCI, if the patient had not already been pre-treated with oral dual-antiplatelet therapy.

Use of stents, type of stents and stent implantation techniques, as well as the use of directional or rotational atherectomy, intravascular ultrasound, and glycoprotein IIb/IIIa inhibitors, was left to the discretion of the operators.

## Study endpoints

The primary study endpoint was periprocedural MI defined as CK-MB mass value ≥ 3 times the upper limit normal or an increase of 50 % of baseline value if already elevated, but stable or falling, at the time of the procedure. The secondary study endpoint was a periprocedural increase in troponin I ≥ 3 times the upper limit normal or an increase of 50 % of the preprocedural value, if >0.04 ng/ml. In addition, the most recent definition of periprocedural MI as a troponin I increase of 5 times the upper limit normal or 20 % of a baseline elevated value was also used.

## Statistical analysis

Statistical analysis was performed using the SPSS 17.0 statistical package. Patients were divided into quintiles according to NLR values (<1.7; 1.7–2.19; 2.20–2.89; 2.9–4.19; ≥4.2). Continuous data are expressed as mean ± SD and categorical data as percentages. Analysis of variance and the chi-square test (or Fisher’s test) were used for continuous and categorical variables, respectively. Multiple logistic regression analysis was performed to evaluate the relationship between NLR and periprocedural myocardial necrosis or infarction, after correction for clinical and angiographic significant differences, which were entered in the model in block. Receiver-operating characteristic (ROC) curve analysis was performed to identify the best predicting value for periprocedural MI.

## Results

**Fig. 1:**
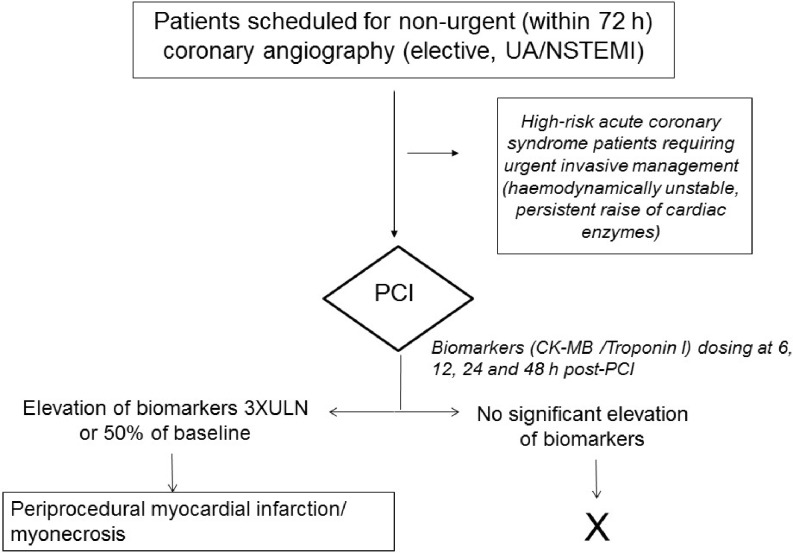
Flow chart for patient inclusion and study procedures

Our population consisted of 1542 patients; 1084 were diagnosed with acute coronary syndrome, while 458 were stable patients scheduled for PCI. The flowchart for patient selection is shown in Fig. 1.

As shown in Tab. 1, higher NLR was related to age (*p* < 0.001), hypertension (*p* = 0.02), hypercholesterolaemia (*p* < 0.001), acute coronary syndrome at admission (*p* = 0.003), use of diuretics (*p* = 0.001), glycaemia, creatinine, fibrinogen, C‑reactive protein and white blood cell count (*p* < 0.001), and inversely with smoking (*p* = 0.001), previous PCI (< 0.001), treatment with beta-blockers, statins and acetylsalicylic acid and haemoglobin values (*p* < 0.001).

**Tab. 1:** Clinical and demographic characteristics neutrophil/lymphocyte ratio quintiles

Baseline clinical characteristics	I quintile *n* = 309	II quintile *n* = 297	III quintile *n* = 320	IV quintile *n* = 307	V quintile *n* = 309	*p* value
Age (mean ± SD)	64.7 ± 10.8	66.9 ± 10.8	66.6 ± 11.3	69.2 ± 10.6	70.5 ± 11.3	<0.001
Male sex (%)	76	76.6	79	77.2	73.7	0.54
Arterial hypertension (%)	70.4	74.9	75.2	70.1	72.4	0.02
Diabetes mellitus (%)	38.6	32.6	38.8	36.3	35.3	0.68
Hypercholesterolaemia (%)	64.6	63	63.5	53.8	46.8	<0.001
Smokers (%)						0.001
Active smokers	26.9	26.2	26.3	23.6	20.5	
Previous smoker	27.7	27	28.6	19.9	21.5	
Renal failure (%)	20.3	22.8	21.9	23.2	25.8	0.09
History of MI (%)	27.2	29.1	27.2	24.5	23.6	0.1
Previous PCI (%)	35.4	33.3	30	27	24.6	<0.001
Previous CABG (%)	11.6	15.4	13.7	14.8	12.6	0.82
Indication to angiography (%)						0.003
Stable angina	32.4	30.1	31.4	19.4	8.9	
Acute coronary syndrome	63.2	62.7	62	76.9	84.6	
LV dysfunction/Arrhythmia	4.4	7.1	6.6	3.8	6.5	
**Therapy at admission**						
ACE inhibitors (%)	44.1	42.9	44.4	46.2	39.6	0.5
ARB (%)	23.4	24.7	26.4	23.1	22	0.55
Beta blockers (%)	65.8	63.2	59	60.1	46.9	<0.001
Nitrates (%)	40.8	44.8	49.1	49.3	33.2	0.23
Calcium antagonists (%)	24.3	25.2	28.6	22.6	21.5	0.30
Diuretics (%)	26.8	23.8	32.2	33	36.3	0.001
Statins (%)	68.8	63.9	61.8	57.4	40.9	<0.001
Acetylsalicylic acid (%)	74.9	73.6	71.5	63.5	51.6	<0.001
Clopidogrel (%)	29	34	30.7	35.6	22.4	0.17
**Main chemistry**						
Glycaemia (mg/dl ± SD)	128.3 ± 50.7	122.2 ± 46.8	129.7 ± 58	131.9 ± 49.6	148.4 ± 65.5	<0.001
Creatinine (mg/dl ± SD)	0.99 ± 0.56	1.06 ± 0.67	1.11 ± 0.73	1.09 ± 0.67	1.33 ± 1.17	<0.001
Platelets (10^3^/ml; mean ± SD)	210.6 ± 58	213.3 ± 53	210.6 ± 58.8	213.4 ± 63.5	217.7 ± 82.9	0.52
Haemoglobin (g/d ± SD l)	13.7 ± 1.6	13.7 ± 1.5	13.5 ± 1.7	13.2 ± 1.8	12.9 ± 2	<0.001
WBC (10^3^/ml; mean ± SD)	7.3 ± 2.8	7.3 ± 1.9	7.5 ± 1.9	8.3 ± 2.4	10.2 ± 3.5	<0.001
HbA1c (mmol/l ± SD)	47.6 ± 15.4	45.6 ± 13.7	46.1 ± 13.1	45.6 ± 12.7	45.7 ± 14.7	0.32
HDL cholesterol (mg/dl)	39.6 ± 11.1	39.5 ± 11.2	40.4 ± 11.6	38.8 ± 11.5	39.2 ± 12.1	0.38
Total cholesterol (mg/dl ± SD)	160.7 ± 42.3	160.6 ± 43.4	160.8 ± 40.2	161.5 ± 43.8	156.9 ± 40.7	0.61
Fibrinogen (mg/dl ± SD)	399 ± 116	418 ± 127	420 ± 117	461 ± 165	479 ± 193	<0.001
C-reactive protein (mg/dl ± SD)	0.52 ± 0.1	0.78 ± 0.19	0.86 ± 0.2	1.53 ± 0.15	3 ± 0.49	<0.001

Tab. 2 displays the main angiographic characteristics and procedural features. NLR was associated with more severe coronary artery disease (*p* = 0.009), tighter stenosis (*p* < 0.001), coronary calcifications (*p* = 0.005), intracoronary thrombus or need for thrombectomy (*p* < 0.001), TIMI flow pre- and post-PCI (*p* < 0.001), and inversely with restenosis (*p* = 0.04) and use of drug-eluting stent (DES) (*p* = 0.001).

**Tab. 2:** Angiographic and procedural characteristics neutrophil/lymphocyte ratio quintiles

Procedural features	I quintile *n* = 498	II quintile *n* = 478	III quintile *n* = 490	IV quintile *n* = 461	V quintile *n* = 461	*p* value
Severe CAD (%)^a^	25	32	32.7	28.8	37.9	0.009
Multivessel disease (%)	54.4	63	56.8	56	63.7	0.17
GP IIb/IIIa inhibitors ^a^	44.3	43.3	39.8	47.2	43.1	0.78
Clopidogrel bolus > 6 h pre-PCI ^a^	21.5	22.4	25.7	25.7	16.5	0.1
Multivessel PCI ^a^	23.2	25.6	27.4	22.9	23.5	0.79
Lesion length (mm ± SD)	22 ± 13.7	20.8 ± 13.7	21.6 ± 13.7	21.5 ± 12.9	21.4 ± 14.8	0.82
Target vessel diameter (mm ± SD)	3 ± 0.6	3 ± 0.6	3 ± 0.6	3 ± 0.6	3 ± 0.6	0.88
% Stenosis (± SD)	89.7 ± 9.5	87.8 ± 10.2	88.8 ± 10	90.4 ± 9.3	90.6 ± 9.5	<0.001
**Target vessel**						0.65
Right coronary artery (%)	21	18.9	22.6	22.5	20.4	
Left main (%)	3	2	3	1.1	1.9	
Left anterior descending (%)	29.6	31.1	25.6	28.1	27.2	
Circumflex branch (%)	16.1	16	16.7	13.4	16.4	
Saphenous venous graft (%)	2.6	4.2	3	2.8	2.7	
Anterolateral branch (%)	11.1	11.8	13.2	13.4	11.6	
Type C lesions (%)	31.4	25.1	29.9	32.4	33.8	0.12
Eccentric plaques (%)	97.9	98.9	97.6	98.1	97.9	0.69
Calcifications (%)	10	14	18.5	17.8	16.2	0.005
Thrombus (%)	6.9	3.4	6.5	14.8	17.5	<0.001
TIMI flow pre-PCI <3 (%)	24.3	21.3	22.6	26.5	33.2	<0.001
In-stent restenosis (%)	8.2	8	5.5	5.1	5.5	0.04
Chronic occlusion (%)	7.9	6.6	9.4	7.1	6	0.42
Bifurcations (%)	23.9	26.6	28.1	20.6	20.4	0.06
Pre-dilatation (%)	64.9	64.4	62.1	72.7	64.6	0.32
Direct stenting (%)	28.5	28	28.7	20.5	27.8	0.18
Drug-eluting stents (%)	71.2	65.7	65.8	62.4	59.9	0.001
Max inflation (atm ± SD)	21.6 ± 3.7	21 ± 3.5	20.9 ± 4.1	20.7 ± 3.6	21 ± 3.4	0.06
Kissing balloon (%)	18.2	14.3	16.6	14.6	11.6	0.05
Thrombectomy (%)	2.1	1.7	2.4	3.6	7.4	<0.001
TIMI post PCI <3 (%)	20.8	21.8	19.3	22.5	26.7	0.003
Any dissection (%)	1.3	1.4	1.8	1.1	0.6	0.35
Slow flow (%)	2.6	1.4	1.5	2.2	4.2	0.12
Coronary perforation (%)	1.3	2.2	0.8	1.1	0.8	0.27
Distal embolisation (%)	1	0.6	1.5	1.4	2	0.16
Additional stent required (%)	1	1.1	2.2	1.6	2	0.24
Side branch loss (%)	0.8	0.8	0.7	1.1	0	0.36

^a^Per patient definition

**Fig. 2:**
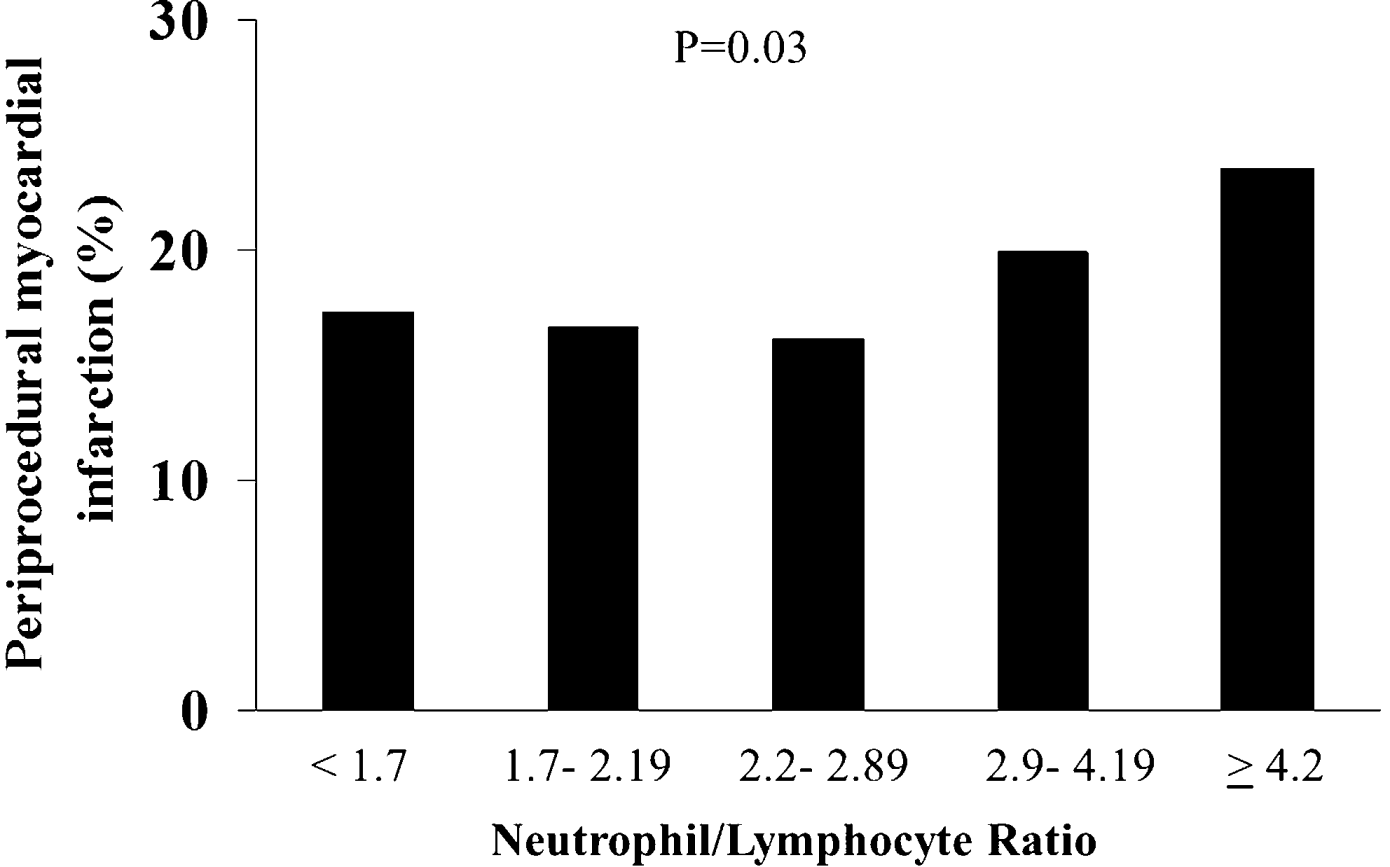
Bar graph showing the prevalence of periprocedural myocardial infarction according to neutrophil to lymphocyte ratio (NLR) quintiles values

**Fig. 3:**
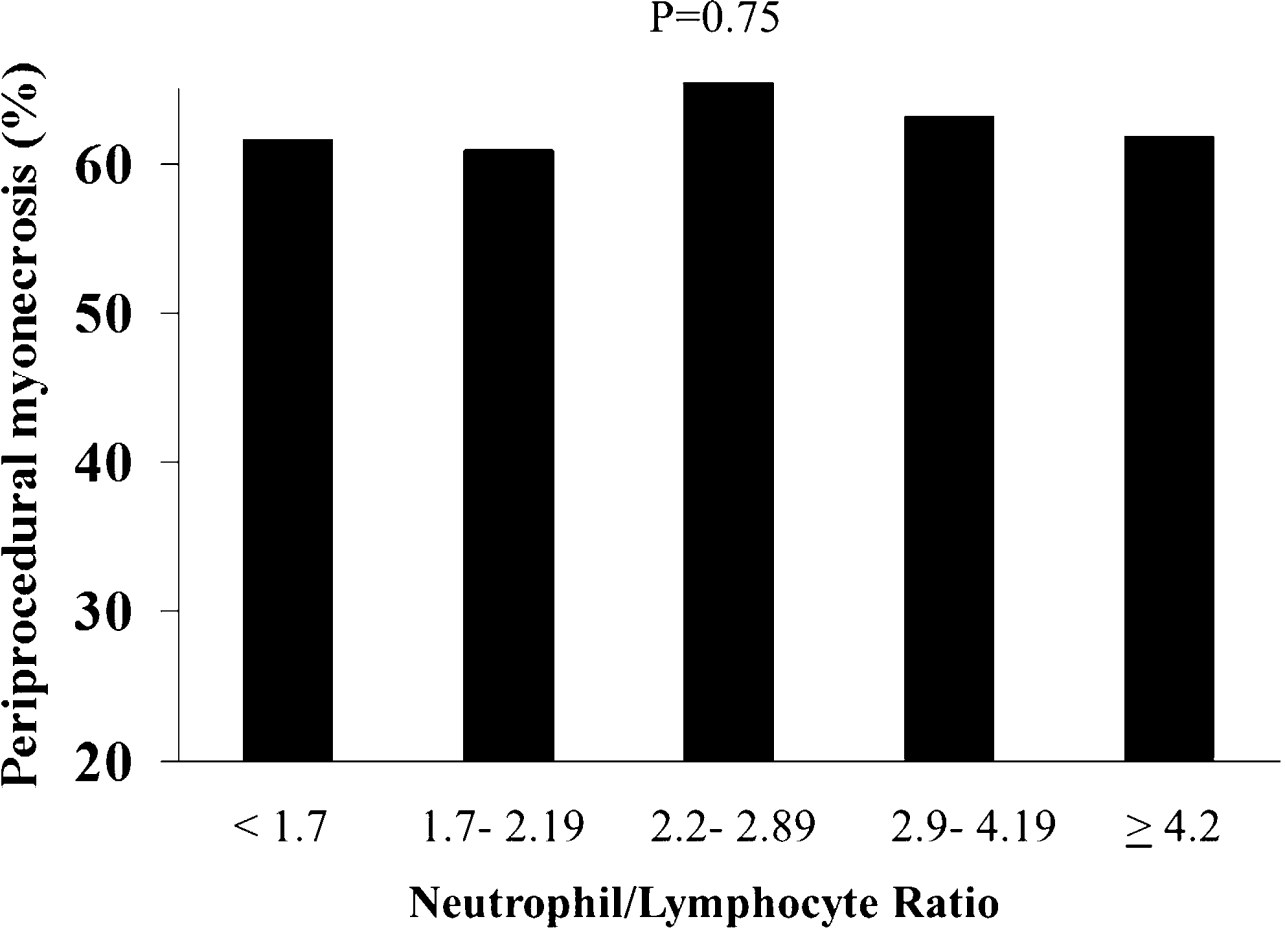
Bar graph showing the prevalence of periprocedural myonecrosis according to neutrophil to lymphocyte ratio (NLR) quintiles values

NLR was associated to a higher occurrence of periprocedural MI (17.3 vs 16.6 vs 14.1 vs 19.9 vs 23.5 %, *p* = 0.03, OR (95 % CI) = 1.11 (1.01–1.22), *p* = 0.03), as displayed in Fig. 2, but did not influence the occurrence of myonecrosis (61.5 vs 60.9 vs 66.6 vs 63.2 vs 61.8 %, *p* = 0.75; OR (95 % CI) = 1.01 (0.94–1.09), *p* = 0.75; Fig. 3).

The results were maintained after correction for baseline differences (age, hypertension, hypercholesterolaemia, acute coronary syndrome, treatment with beta-blockers, statins, acetylsalicylic acid and diuretics, glycaemia, creatinine, fibrinogen, C‑reactive protein, haemoglobin, white blood cell count, smoking, previous PCI, severity of coronary artery disease, % stenosis, coronary calcifications, intracoronary thrombus, thrombectomy, TIMI flow pre- and post-PCI, restenosis and use of DES), for both myonecrosis (adjusted OR (95 % CI) = 0.99 (0.63–1.54), *p* = 0.96), and periprocedural MI (adjusted OR (95 % CI) = 1.33 (1.02–2.3), *p* = 0.02) and also when considering NLR as a continuous variable (myonecrosis: adjusted OR (95 % CI) = 1.01 (0.97–1.06), *p* = 0.63; periprocedural MI: adjusted OR (95 % CI) = 1.05 (1.001–1.10), *p* = 0.05).

**Fig. 4:**
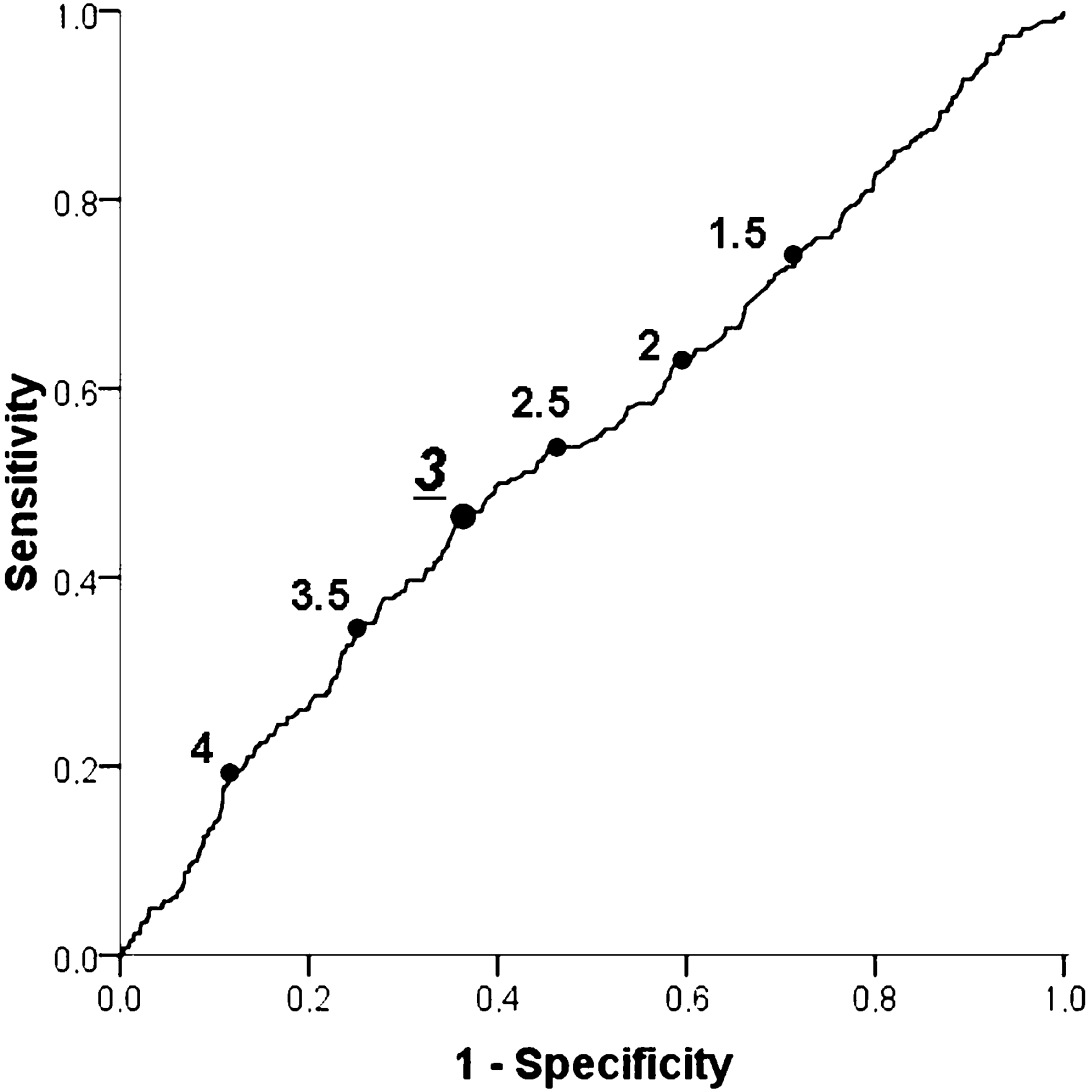
Receiver-operating characteristic (ROC) curve for the neutrophil to lymphocyte ratio (NLR) values in the prediction of periprocedural myocardial infarction

**Fig. 5:**
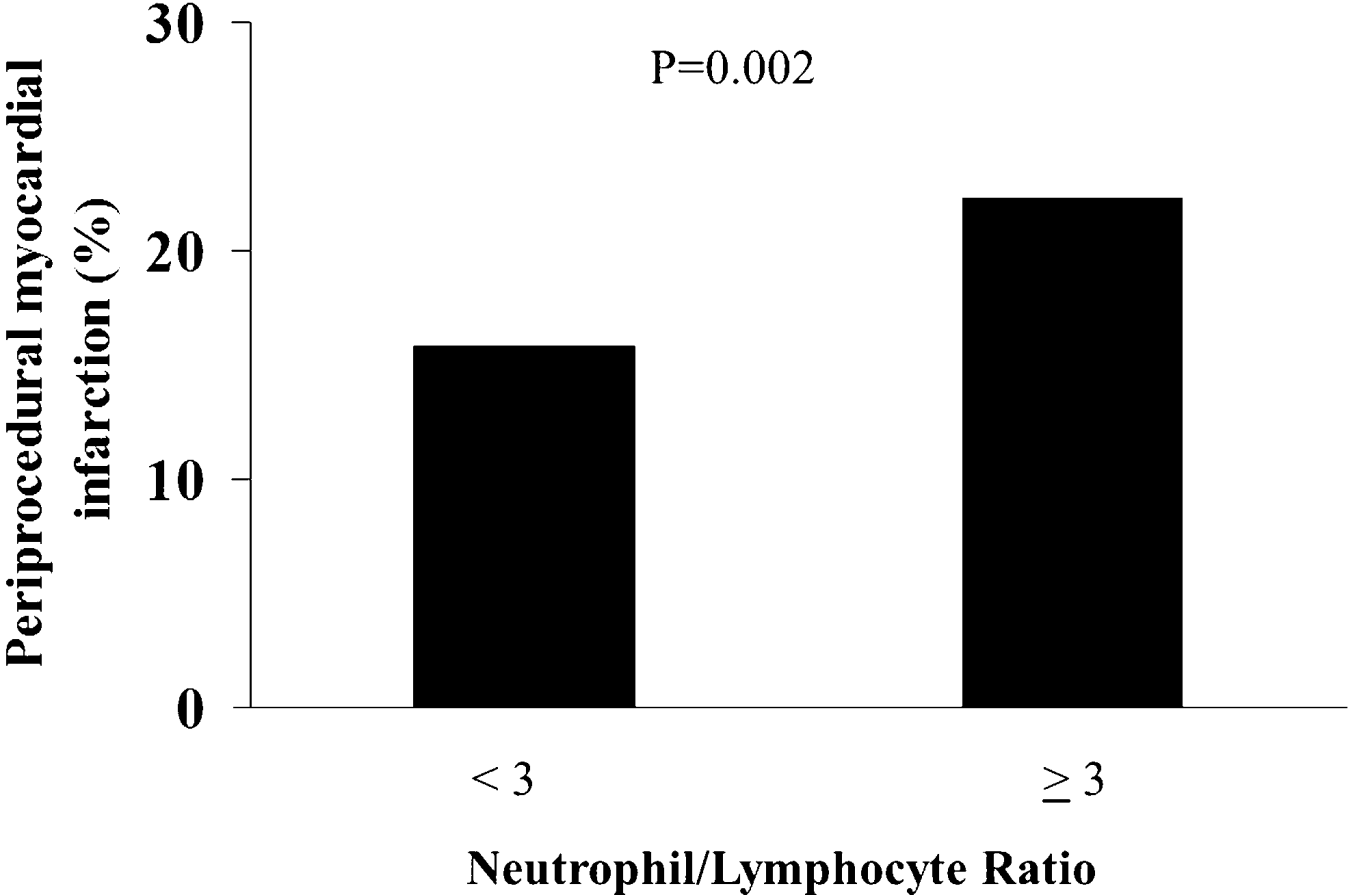
Bar graph showing the prevalence of periprocedural myocardial infarction in patients with neutrophil to lymphocyte ratio (NLR) ≥ 3

At ROC curve analysis, NLR ≥ 3 proved to have the best predictive value for the risk of periprocedural MI (AUC 0.544, *p* = 0.03, Fig. 4), and in fact, when dividing our patients for NLR < or ≥ 3, a higher rate of periprocedural MI was found in patients with elevated NLR (15.8 vs 22.3 %, *p* = 0.02, adjusted OR (95 % CI)  = 1.39 (1.03–1.88), *p* = 0.03, as shown in Fig. 5).

Analogous results were achieved when separately considering acute coronary syndrome (*n* = 1084) and stable patients (*n* = 458). In fact, among acute patients, an increased rate of periprocedural MI was observed for NLR ≥ 3 (periprocedural MI: 28.1 vs 20.7 %, *p* = 0.03; adjusted OR (95 % CI)  = 1.53 (1.01–2.3), *p* = 0.04; myonecrosis: 57.6 vs 56.6 %, *p* = 0.82; adjusted OR (95 % CI) = 1.05 (0.67–1.63), *p* = 0.84). On the contrary, among patients with stable coronary artery disease, we confirmed a similar occurrence of myonecrosis according to NLR < or ≥ 3 (70.3 vs 67.5 %, *p* = 0.48; adjusted OR (95 % CI) = 1.21 (0.63–2.31), *p* = 0.57) whereas we observed an absolute increase in the rate of periprocedural MI (19.2 vs 13.7 %, *p* = 0.22), although not reaching statistical significance at multivariate analysis (adjusted OR (95 % CI) = 2.2 (0.94–4.9), *p* = 0.07).

Results did not change when applying a more recent definition of periprocedural MI (troponin I > 5 x upper limit normal or 20 % of baseline value), with a trend for higher periprocedural myocardial injury in patients with NLR ≥ 3 (67.5 vs 64.6 %, *p* = 0.21; adjusted OR (95 % CI) = 1.21 (0.92–1.58), *p* = 0.16).

## Discussion

This is the first study to evaluate the role of NLR on periprocedural MI in patients undergoing non-urgent PCI. Our main finding is that higher NLR increases the risk of periprocedural MI; this applies especially for NLR ≥ 3.

In the last decades, various efforts have been made to improve the outcome in patients with coronary artery disease, by shortening the time to treatment in acute myocardial infarction and introducing new techniques for the percutaneous treatment of more and more complex lesions [15–17]. However, despite the great reduction in mortality observed, especially in the setting of STEMI, suboptimal results have still been obtained in certain subsets of high-risk patients [18–21]. Therefore, in recent years, great efforts have been focused on the prevention of periprocedural complications. In effect, periprocedural myocardial injury has been shown by MRI studies to occur in the majority of PCI procedures [22], thus requiring the identification of new biomarkers to improve risk stratification.

Inflammation is a well-established risk factor for coronary artery disease, playing a role in the progression of atherosclerosis and regulating the process leading to the “instabilisation” of a plaque, and then to an acute ischaemic event [23]. Moreover, inflammatory response has been strictly linked to an enhanced pro-thrombotic status, by increasing the circulating levels of fibrinogen and other coagulative factors and by enhancing platelet reactivity through reactive oxygen species, cytokines and other mediators [24]. Many studies have evaluated the prognostic role of inflammatory biomarkers in cardiovascular disease, with contrasting results [25]. Initial studies showed that an elevated total white blood cell count was associated with increased mortality and worse outcomes after acute MI, and mainly for patients with higher neutrophil counts [26]. More recently, attention shifted to the NLR, combining the effects of the nonspecific inflammatory response, mediated by neutrophils, and the subsequent regulatory immune response, involving lymphocytes.

Indeed, neutrophils have been claimed to be responsible for each step leading to acute coronary events and in addition, a low lymphocyte count has also been associated with worse prognosis in patients with ischemic heart disease and unstable angina [27], as a certain subset of lymphocytes has been shown to play an inhibitory role in atherosclerosis, possibly by controlling and regulating the inflammatory response [28].

In fact, NLR has been associated with indirect indicators of atherosclerosis, such as the coronary calcium score, and to the extent of angiographically defined coronary artery disease [8]. Moreover, NLR has emerged as a predictor of clinical outcome in both stable patients undergoing percutaneous or surgical coronary revascularisation [29] and in patients with acute coronary syndrome [30], with the majority of studies conducted in the setting of acute myocardial infarction. In particular, Ergelen et al. reported in a large cohort of STEMI patients that NLR levels >6.97 were associated with increased in-hospital and long-term cardiovascular mortality, and similar results were reported in a Korean population of patients undergoing primary PCI [11, 31]. However, so far few studies have evaluated the role of NLR in patients undergoing elective percutaneous coronary revascularisation.

In our study we observed a relevant impact of elevated NLR on periprocedural MI in a large population of patients undergoing elective PCI, as all acute patients underwent pharmacological stabilisation before coronary angiography. Indeed, confirming previous findings, higher NLR has been associated with acute coronary syndrome, intracoronary thrombus and other inflammatory parameters, such as C‑reactive protein and fibrinogen; however, our positive association between NLR and periprocedural MI was maintained at multivariate analysis even after correction for potential confounders. Moreover, using the ROC curve we identified NLR ≥ 3 as being best for predicting the risk of periprocedural MI.

Similar results were obtained by Akpet and colleagues, although in a population of STEMI patients, reporting that an NLR > 3.3 had a 74 % specificity and 83 % sensitivity to predict the phenomenon of no-reflow [32], an event that has been associated to a larger infarct size and a worse prognosis in patients undergoing primary PCI.

Analogous results were reported by Soylu et al. linking NLR to the no-reflow development in patients undergoing primary PCI [33]. In addition, Yilmaz et al. identified a significant association between this inflammatory marker and the presence of intracoronary thrombus [10], which has been demonstrated to increase the risk of distal embolisation and impaired myocardial perfusion in patients undergoing PCI. In this context, the extensive use of potent antiplatelet drugs, such as abciximab and other GP IIb/IIIa inhibitors in our patients, could have influenced thrombus burden and then prevented a large number of events, although no difference in administration of GP IIb/IIIa inhibitors was observed across the NLR quintiles.

Therefore, future studies are certainly needed to evaluate whether lowering NLR can prevent periprocedural myocardial injury after PCI and whether these effects can contribute to the myocardial protection proposed for certain drugs, such as for acetylsalicylic acid and especially for statins [34, 35]. In fact, in our study they were significantly inversely related to NLR, an association that can be explained by the pleiotropic, anti-inflammatory effects of statins, rather than their hypolipaemic effect.

## Study limitations

A first limitation to our study can be considered the inclusion of a cohort of patients who were heterogeneous for certain clinical baseline characteristics, including risk factors as older age, hyperglycaemia, acute presentation or previous revascularisation, which are known per se to influence the occurrence of periprocedural MI. We observed a relatively high rate of periprocedural MI, which could be explained by the large number of complex, high-risk patients, including those with acute coronary syndrome. The lower rate of events among stable patients could have contributed to the lack of significance of the association between NLR and periprocedural MI in this context.

However, the aim of the present study was to assess the impact of NLR in a real-life population of patients undergoing PCI and in addition, the independent relationship of NLR and periprocedural MI was confirmed even after correction for potential confounders. Even though the greatest increase in periprocedural MI was observed in patients with NLR in the IV and V quintiles, especially for values above 3, as identified by the ROC curve analysis, no linear relationship was observed between NLR and periprocedural MI. Nevertheless, the association for this parameter with NLR was quite weak, and certainly its impact in clinical practice needs further confirmation.

In fact, we did not collect data from long-term follow-up in our patients and therefore we cannot evaluate the long-term prognostic impact of periprocedural myonecrosis and effects of NLR on outcome after stent implantation. However, it has been advocated that periprocedural MI is probably only of minor relevance for patient prognosis, and especially for troponin elevation. The frequent increase in troponin after PCI could have potentially masked the relationship with NLR, thus rendering its determination non-specific.

In addition, we did not evaluate NLR changes after PCI, although very modest changes in these parameters are usually observed in elective patients; this in contrast to STEMI patients where the leukocyte profile at 24 hours after primary PCI, but not the baseline haematological indexes, has been demonstrated to be an independent predictor of cardiovascular outcome [8].

Finally, the use of intravascular imaging, as intravascular ultrasound or optical coherence tomography, would certainly have prevented periprocedural complications in patients with suboptimal procedural results after coronary stent implantation.

## Conclusions

The present study is the first to demonstrate that in patients undergoing non-urgent PCI a higher NLR increases the risk of periprocedural myocardial infarction, especially for values ≥ 3.
